# A276 A SYSTEMATIC REVIEW OF THE ASSOCIATION BETWEEN EOSINOPHILIC ESOPHAGITIS AND INFLAMMATORY BOWEL DISEASE

**DOI:** 10.1093/jcag/gwad061.276

**Published:** 2024-02-14

**Authors:** R P Yanofsky, R Jogendran, T Hoxha, A Pathak, A Orchanian-Cheff, P Tandon

**Affiliations:** Gastroenterology and Hepatology, University of Toronto, Toronto, ON, Canada; Gastroenterology and Hepatology, University of Toronto, Toronto, ON, Canada; Gastroenterology and Hepatology, University of Toronto, Toronto, ON, Canada; Michigan State University College of Osteopathic Medicine, East Lansing, MI; University Health Network, Toronto, ON, Canada; University Health Network, Toronto, ON, Canada

## Abstract

**Background:**

Inflammatory bowel disease (IBD) and eosinophilic esophagitis (EoE) are chronic inflammatory conditions of the gastrointestinal tract. IBD and EoE have been linked to other autoimmune conditions. There has been conflicting evidence on whether these two diseases are associated.

**Aims:**

The purpose of the current study is to perform a systematic review evaluating the incidence of co-existing IBD and EoE. To determine whether the relationship between IBD and EOE is unique for autoimmune conditions, we also evaluated the relationship between other autoimmune conditions such as rheumatoid arthritis and atopic dermatitis and EOE.

**Methods:**

A comprehensive search strategy was conducted on July 10, 2023 using MESH terms for eosinophilic esophagitis and IBD, autoimmune disorders or connective tissue disorders in Ovid MEDLINE; Ovid Embase; Cochrane Database of Systematic Reviews (Ovid); and Cochrane Central Register of Controlled Trials (Ovid). The primary outcome was defined as the frequency of concomitant IBD and EOE compared to the frequency of IBD alone. Secondary outcomes included the frequency of concomitant autoimmune conditions and EOE compared the frequency of the autoimmune condition alone, as well as the examination of clinical, biomarker, and endoscopic outcomes of patients with concomitant IBD and EOE compared to patients with IBD alone.

Five independent reviewers participated in study selection applying eligibility criteria and selecting studies for inclusion. Duplicate items were removed and title and abstracts screening occurred. Any publication deemed potentially relevant by was carried forward to the full-text review. Discrepancies were resolved by consensus.

**Results:**

Our systematic search identified 2144 potential studies. After screening titles, we were left with 268 studies. Following full text review, 50 studies remained which included 9 articles and 19 abstracts on IBD and EOE, and 8 articles and 14 abstracts on other autoimmune conditions and EOE.

The 9 articles on IBD and EOE consisted of 6 retrospective cohort, 1 prospective cohort, 1 case-control and 1 case series study. 8 of the studies were conducted in the United States and 1 was conducted in Italy. There were 4 studies on adult populations, 4 studies on pediatric populations, and 1 study in both. There was a total of 133,451,249 patients evaluated, the majority being from one study (n=133,308,339 patients). The number of concomitant IBD and EOE patients per study ranged from 5 to 3506 patients and the number of IBD patients without EOE ranged from 33 to 701,601 patients. In the general population, the number of EOE patients alone per study ranged from 33 to 102,048. Completion of data extraction and statistical analysis is ongoing.

**Conclusions:**

There may be an association between IBD and EOE. Further data analysis is ongoing.

**Funding Agencies:**

None

